# AGE-FRIENDLY HEALTH SYSTEMS 4MS IMPLEMENTATION: THE NEED FOR BOOSTER STRATEGIES

**DOI:** 10.1093/geroni/igae098.0477

**Published:** 2024-12-31

**Authors:** Anne Pohnert, Nicholas Schiltz, Mary Dolansky

**Affiliations:** CVSHealth, Bellingham, Massachusetts, United States; Case Western Reserve University, Cleveland, Ohio, United States; Case Western Reserve University, Cleveland, Ohio, United States

## Abstract

This study describes the implementation of the Age-Friendly Health System (AFHS) 4Ms Framework within the largest convenient care health system in the U.S. Implementation and improvement science methods were used to facilitate adoption of the 4Ms into ~1,100 MinuteClinics located in 35 states and DC, and to educate the ~3,300 providers working at these locations. Modifications to the electronic health record enabled the comprehensive documentation of assessments and actions related to the AFHS 4Ms. Formative evaluation utilized Tableau dashboards for provider and manager reporting. In the initial year, the project employed a quality improvement design. Only 22% delivery of the 4Ms was achieved after the first year indicating that “booster” strategies were needed. Following 18 months of implementing additional booster strategies to enhance 4Ms uptake, the system received IHI Regional Committed to Care Excellence recognition, achieving significant improvements across all 4Ms. As of 2024 Q1, 38.0% of eligible patients visits receive all 4Ms, while XX% receive at least one of the 4Ms. While improvement and implementation science methods facilitated initial implementation, further understanding and enhancement strategies are essential for scaling up and widespread adoption.

